# Association of Driver Oncogenic Alterations with SUVmax, Preoperative Serum Calcium, and Smoking Status in Surgically Resected Non-Small-Cell Lung Cancer: A Retrospective Single-Center Study

**DOI:** 10.3390/jcm15114100

**Published:** 2026-05-26

**Authors:** Nikolaos Korodimos, Małgorzata Edyta Wojtyś, Konstantinos Kostopanagiotou, Ilias Santaitidis, Ioannis Tomos, Periklis Foukas, Konstantinos Kontzoglou, Anna Koumarianou, Sofoklis Mitsos, Anastasios Moisiadis, Periklis Tomos

**Affiliations:** 1 University Thoracic Surgery Clinic, Attikon University General Hospital, 12462 Athens, Greece; korodimos@yahoo.com (N.K.); kostop@hotmail.co.uk (K.K.); uni.drhgs@gmail.com (I.S.); sophocmit@yahoo.gr (S.M.); anas.mois@gmail.com (A.M.); periklistomos@hotmail.com (P.T.); 2 Department of Thoracic Surgery and Transplantation, Pomeranian Medical University in Szczecin, 70-891 Szczecin, Poland; 3 Pulmonary Department, “Sotiria” Athens Hospital for Thoracic Diseases, 11527 Athens, Greece; etomos@hotmail.com; 4 University Laboratory of Histopathology, Attikon University General Hospital, 12462 Athens, Greece; pfoukas@yahoo.com; 5 University Surgical Clinic, Laiko General Hospital of Athens, 11527 Athens, Greece; kckont@med.uoa.gr; 6 University Fourth Department of Internal Medicine, Attikon University General Hospital, 12462 Athens, Greece; akoumari@yahoo.com

**Keywords:** non-small-cell lung cancer, *KRAS*, *EGFR*, *ROS1*, *ALK*, PD-L1, *BRAF*, SUVmax, PET/CT, serum calcium, smoking

## Abstract

**Background:** Non-small-cell lung cancer (NSCLC) is driven by distinct oncogenic alterations with important therapeutic and prognostic implications. Noninvasive biomarkers that predict molecular status in surgically resectable disease may aid in their management. We investigated the association of preoperative primary-tumor SUVmax on PET/CT, smoking history, and corrected serum calcium levels with driver oncogenic alterations and PD-L1 expression in surgically resected NSCLC. **Methods:** We retrospectively studied 170 patients with surgically resected NSCLC at a single tertiary center. Resected tumors were assessed for *EGFR*, *KRAS*, and *BRAF* mutations, *ALK* and *ROS1* rearrangements, and PD-L1 expression. Associations between molecular status, PD-L1 expression, and clinicometabolic parameters were evaluated using univariate analyses and multivariable regression models. **Results:** A driver alteration was detected in 51.2% of tumors, and 30% of evaluable cases showed high PD-L1 expression (≥50%). Corrected serum calcium was positively correlated with SUVmax and emerged as the strongest independent predictor retained in the final linear regression model, with pack-years also contributing independently. Most molecular subgroups did not show significant differences in SUVmax. *EGFR*-mutated tumors showed a trend toward a lower SUVmax compared with *EGFR* wild-type tumors, although this did not reach statistical significance. Smoking history was not significantly associated with PD-L1 expression, and pack-years did not differ significantly across the molecular groups examined. **Conclusions:** In this cohort of surgically resected NSCLC, preoperative corrected serum calcium and smoking exposure were more closely associated with tumor metabolic activity than with specific molecular alterations. These findings suggest that simple clinical and biochemical parameters may provide complementary information, although their utility for discriminating individual molecular subgroups appears limited.

## 1. Introduction

Lung cancer is approximately 85% non-small-cell lung (NSCLC) cancer and is the leading cause of cancer-related mortality worldwide. Despite many advances in screening and treatment, many patients are still diagnosed at an advanced stage. This underscores the need for early diagnosis. The diagnosis of NSCLC is based on accurate histological confirmation, as well as tumor staging and molecular analysis. In preoperative staging, 18F-FDG PET/CT plays an important role and helps us evaluate the metabolic activity of the tumor. In recent years, treatment strategies beyond surgical resection and chemotherapy have expanded to targeted therapies based on biomarkers and immune checkpoint inhibitors [1]. This is not only the case in advanced disease but also in the neoadjuvant setting for resectable tumors. In this evolving context, there is growing interest in simple preoperative parameters that can provide complementary information about tumor biology and help improve clinical decision making. Given the heterogeneous clinical course of NSCLC, improved understanding of factors associated with different patterns of disease progression and prognosis remains important for refining patient stratification and individualized management [2]. Previous work has emphasized that NSCLC progression may include distinct patterns, such as lymph node involvement, local or regional recurrence, and distant metastasis, each of which may carry different prognostic implications [2].

In many cases, we encounter oncogenic alterations that can be addressed, such as *EGFR*, *KRAS*, *BRAF* mutations, *ROS1* and *ALK* rearrangements, and differential expression of PDL-1. Immunotherapeutic strategies and targeted therapies can be guided by these alterations [3,4]. Molecular analysis of tumor tissue or liquid biopsy in advanced disease is the standard treatment. In contrast, comprehensive molecular characterization is increasingly recommended in early-stage surgically resectable NSCLC as neoadjuvant targeted therapies and immunotherapies become available [5,6].

In clinical practice, there is considerable interest in simple preoperative indicators that may be associated with tumor metabolic activity, molecular alterations, or PD-L1 expression, including clinical variables such as smoking status, biochemical markers, and imaging parameters. Several studies have shown that *EGFR* mutations are more common in never-smokers, females, and adenocarcinoma histology, whereas *KRAS* mutations are enriched in current or former heavy smokers [7,8,9,10]. The relationship between 18F-fluordeoxyglucose (FDG) positron emission tomography (PET)/computed tomography (CT) metabolic parameters, particularly the maximum standard uptake value (SUVmax), and oncogenic drivers has been explored, but with controversial results [9,11,12,13,14,15]. Some reports suggest that *EGFR*-mutant tumors tend to exhibit lower FDG uptake, whereas others have failed to confirm a consistent association [11,12,13,14].

In addition, alterations in systemic biochemical parameters, such as serum calcium, have been linked to prognosis and metastatic patterns in NSCLC, including hypercalcemia in squamous histology and low calcium levels as a marker of unfavorable outcome and bone metastasis [16,17]. Whether serum calcium is related not only to disease burden and prognosis but also to specific molecular subtypes is less clear, particularly in the context of surgically resectable disease.

The aim of the present study was to evaluate the association of the preoperative SUVmax of the primary tumor on PET/CT, preoperative serum calcium, and smoking status with driver oncogenic alterations and PD-L1 expression in a cohort of patients with surgically resected NSCLC. We evaluated whether simple preoperative metabolic, biochemical, and clinical parameters can provide complementary information about tumor metabolic activity and molecular profile in operable non-small-cell lung cancer (NSCLC).

## 2. Materials and Methods

### 2.1. Study Design and Patient Population

This was a retrospective single-center cohort study of patients who underwent curative-intent surgical resection for histologically confirmed NSCLC at the University Thoracic Clinic of Attikon University General Hospital of Athens between 2019 and 2024. Histologically confirmed NSCLC; lobectomy, bilobectomy, segmentectomy, or pneumonectomy with systematic lymph node dissection; 18F-FDG PET/CT completed at our institution within 4 weeks before surgery; and formalin-fixed paraffin-embedded (FFPE) tumor tissue molecular testing and complete clinical information, including smoking history and preoperative serum calcium, were the inclusion criteria. Patients who had small-cell lung cancer, mixed small-cell histology, previous neoadjuvant systemic therapy, or recurring disease, and those having incomplete data, were excluded. Patients were included only when complete clinical, imaging, laboratory, pathological, and molecular data required for the planned analyses were available. Cases with missing essential data, including missing preoperative serum calcium or albumin values, missing smoking history, unavailable PET/CT-derived SUVmax, or incomplete molecular/PD-L1 assessment, were excluded from the corresponding analyses. Preoperative calcium values were interpreted after albumin correction using a standard correction formula [18], and available medical records were reviewed for major comorbidities or clinical conditions that could substantially affect serum calcium levels. In particular, patients with incomplete laboratory information or insufficient documentation regarding relevant calcium-related clinical factors were not included in the final analytical cohort. Because of the retrospective design, a more detailed assessment of all possible calcium-modifying factors, including medication exposure and subtle endocrine or renal abnormalities, was not uniformly available and is acknowledged as a limitation. The institutional ethics committee accepted the study protocol (approval number: 271/14-5-2021) and implemented it in line with the Declaration of Helsinki. Since the analysis was retrospective, the need to have informed consent was waived.

### 2.2. Clinical and Laboratory Data

Electronic medical records, such as age, sex, performance status, comorbidities, and tumor histology, as well as pathological stage (AJCC 8th edition), and smoking status, were used to collect demographic and clinical data. The smoking history was measured in pack-years, and patients were classified as never-smokers, former smokers (patients who had not smoked at least 12 months before surgery) or current smokers. To analyze them, the ever-smokers were further stratified based on cumulative smoking exposure. where heavy smoking was categorized as 30 pack-years, and light smoking was categorized as <30 pack-years, as commonly used thresholds in lung cancer epidemiology and surveillance studies. Preoperative albumin and serum total calcium levels, 7–14 days prior to surgery, were also checked. Corrected calcium was calculated using the albumin-adjusted calcium formula: corrected calcium (mg/dL) = measured total calcium (mg/dL) + 0.8 × [4.0 − serum albumin (g/dL)] [18]. The institutional reference range for total serum calcium was approximately 8.5–10.5 mg/dL. Calcium and albumin values were obtained from preoperative laboratory tests performed 7–14 days before surgery.

### 2.3. Measurement of SUVmax

Preoperative whole-body 18F-FDG PET/CT was done on all the patients. Attenuation-corrected images of the primary lung lesion were used to measure its SUVmax utilizing a standardized protocol. In brief, the primary tumor and SUVmax, which were automatically recorded inside the workstation software, were a volume of interest that was calculated automatically. In multifocal disease cases, the lesion of interest at the surgical specimen was the dominant lesion. To improve consistency of SUVmax assessment, PET/CT examinations were performed according to the institutional imaging protocol. Patients fasted before tracer administration, and blood glucose levels were checked before 18F-FDG injection according to routine clinical practice. Image acquisition and reconstruction were performed using standardized institutional parameters, and SUVmax was measured on attenuation-corrected images of the primary tumor. However, because this was a retrospective study covering several years, possible inter-instrument and protocol-related variability may have affected SUVmax values. Therefore, SUVmax measurements were interpreted within the known limitations of PET acquisition, reconstruction, and quantitative analysis variability [19,20].

### 2.4. Pathology and Molecular Analysis

Those were all the cases of surgical specimens that were examined by senior thoracic pathologists. The histological subtype and grade were prescribed based on the WHO classification. Formalin-fixed paraffin-embedded (FFPE) tumor tissue from the resected specimens was used for molecular and immunohistochemical analysis. Tumor areas with adequate tumor cellularity were selected by the pathologist before molecular testing. *EGFR* mutation status, including exons 18–21, as well as *KRAS* and *BRAF* mutation status, was assessed using validated molecular methods, including PCR-based sequencing and/or next-generation sequencing (NGS), according to tissue availability and routine institutional practice during the study period [21]. *ALK* and *ROS1* alterations were evaluated as gene rearrangements rather than point mutations. These rearrangements were assessed using immunohistochemistry as a screening method and/or fluorescence in situ hybridization (FISH) for confirmation, according to the diagnostic workflow used during the study period [21]. PD-L1 expression was assessed separately by immunohistochemistry using the 22C3 antibody clone. PD-L1 expression was reported as tumor proportion score (TPS), defined as the percentage of viable tumor cells showing membranous staining. For analysis, PD-L1 TPS was categorized as <1%, 1–49%, and ≥50% [22].

### 2.5. Statistical Analysis

In the present study, different statistical techniques were used depending on the level of measurement of the variables. The χ^2^ test of independence was applied for the nominal variables, with an assessment of the strength of the relationship through the Phi and Cramer’s V indices. Exact tests were also performed due to possible limitations of a small sample. Kendall’s Tau-b and Tau-c coefficients were used for ordinal variables. A *t*-test for independent samples was also applied, after checking the necessary conditions, in cases of comparison of means between two independent groups. The Pearson correlation coefficient was used to examine the relationships between quantitative variables, while linear regression was applied with a stepwise method to investigate predictive relationships. All analyses were performed using IBM SPSS Statistics, version 29.0 (IBM Corp., Armonk, NY, USA), with the Exact Tests module, and the statistical significance level was set at 0.05.

## 3. Results

**Figure 1:**
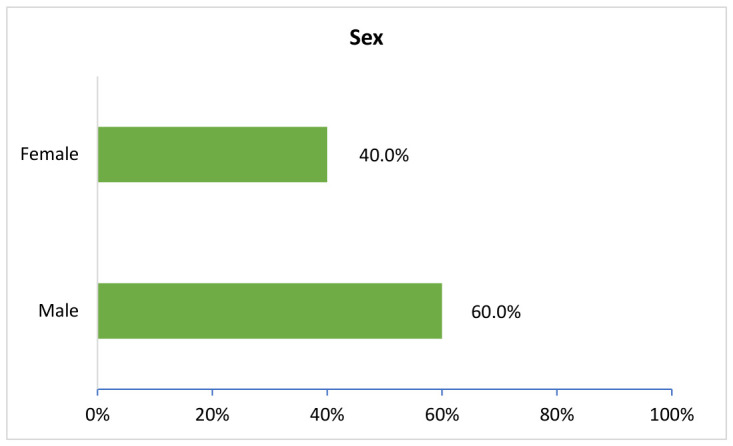
Sex distribution of the study cohort.

This study included 170 patients with a mean age of 66.1 years (SD: 7.52) (Table 1). The median age was 66 years, and the range was 48 to 85 years. Of these, men accounted for the majority of the sample, with a percentage of 60%, and women accounted for 40%. (Figure 1).

**Figure 2:**
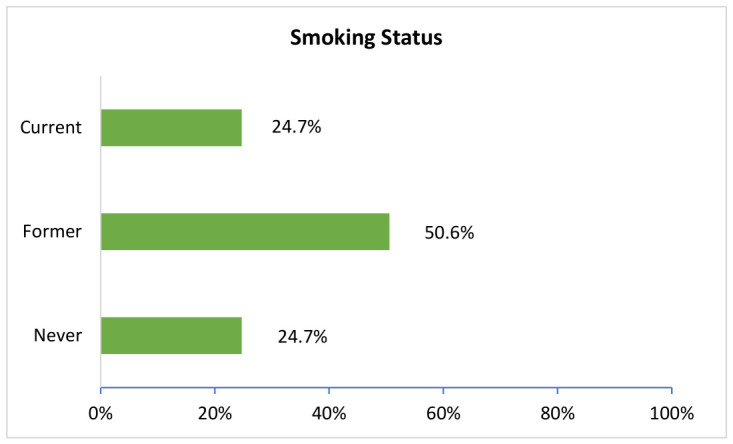
Distribution of smoking status among the study cohort.

Regarding smoking habits, 50.6% were former smokers, while current smokers constituted 24.7% and non-smokers 24.7% as well. The mean smoking burden was 31.9 pack-years (SD: 14.44), with a median value of 36 pack-years, and the values ranged from 0 to 62 pack-years (Figure 2).

**Figure 3:**
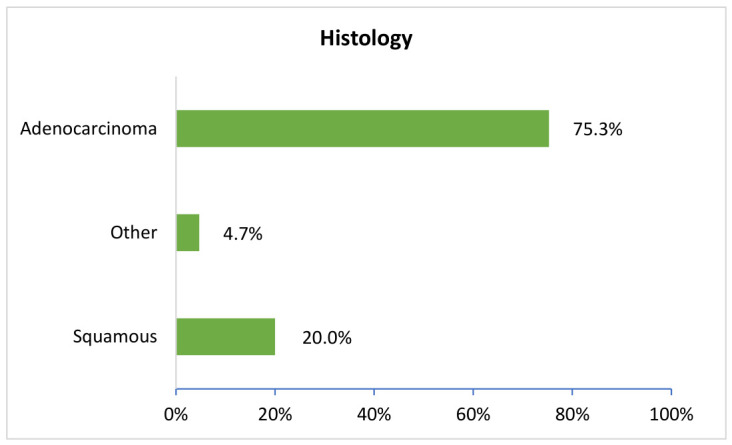
Histological subtype distribution of non-small-cell lung cancer cases.

Tumor histology showed that 128 patients (75.3%) had adenocarcinoma (making adenocarcinoma the most common histological type), 34 patients (20.0%) had squamous-cell carcinoma, and the remaining eight patients (4.7%) had other histological types (Figure 3).

**Figure 4:**
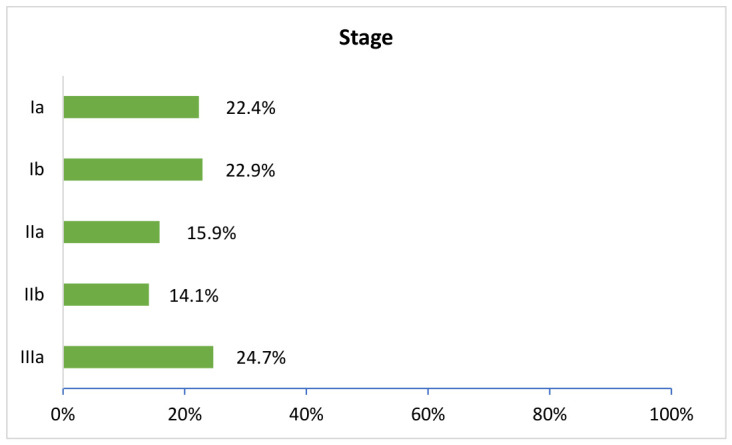
Pathological stage distribution according to the AJCC 8th edition.

Of these, stage Ia had 38 patients (22.4%), stage Ib 39 patients (22.9%), stage IIa 27 patients (15.9%), stage IIb 24 patients (14.1%) and stage IIIa 42 patients (24.7%) (Figure 4).

**Figure 5:**
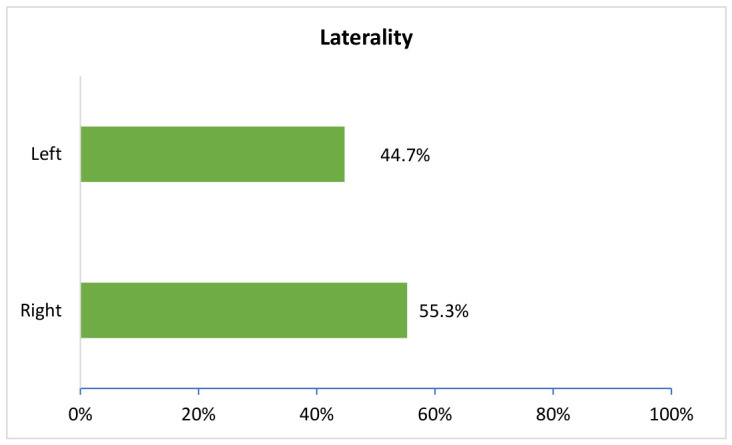
Distribution of tumor laterality.

**Figure 6:**
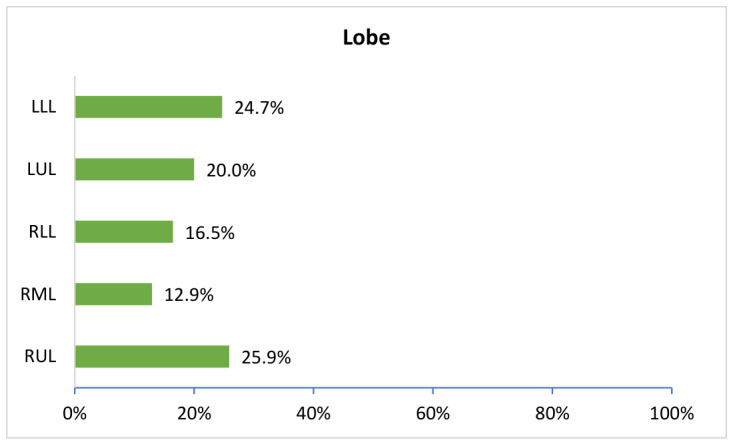
Distribution of primary tumor location by lung lobe.

Regarding tumor location, in 94 patients (55.3%), it was right in 76 patients (44.7%), and the tumor was located in the left lung (Figure 5). Specifically, the most common tumor site was the right upper lobe in 44 patients (25.9%), then in the left lower lobe in 42 patients (24.7%), the left upper lobe in 34 patients (20.0%), the right lower lobe in 28 patients (16.5%) and the right middle lobe in 22 patients (12.9%) (Figure 6).

**Figure 7:**
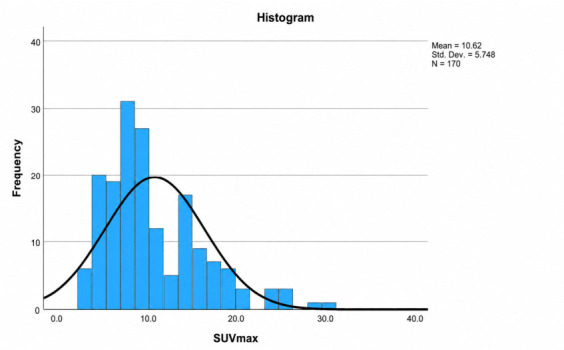
Distribution of primary tumor SUVmax values. Blue bars represent the histogram frequencies, and the black curve represents the fitted normal distribution.

The mean SUVmax was 10.62 (SD: 5.75). The median value was 9, and the range was from 2 to 30.3 (Figure 7). Corrected calcium levels were also evaluated, with a mean value of 9.41 mg/dL (SD: 0.64), a median value of 9.385 mg/dL and a range of values from 8.15 to 11.5 mg/dL (Table 1).

**Figure 8:**
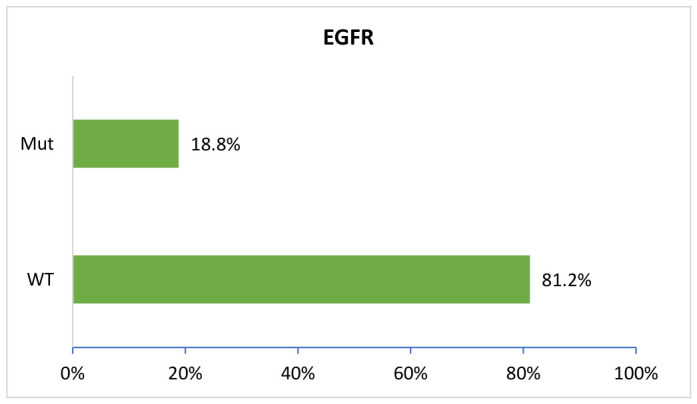
Distribution of *EGFR* mutation status.

**Figure 9:**
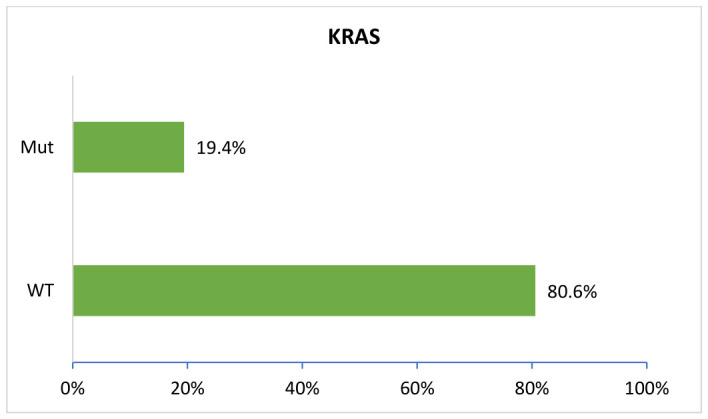
Distribution of *KRAS* mutation status.

**Figure 10:**
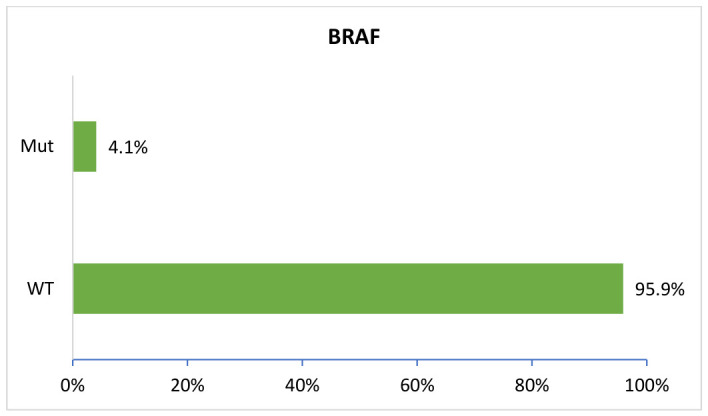
Distribution of *BRAF* mutation status.

**Figure 11:**
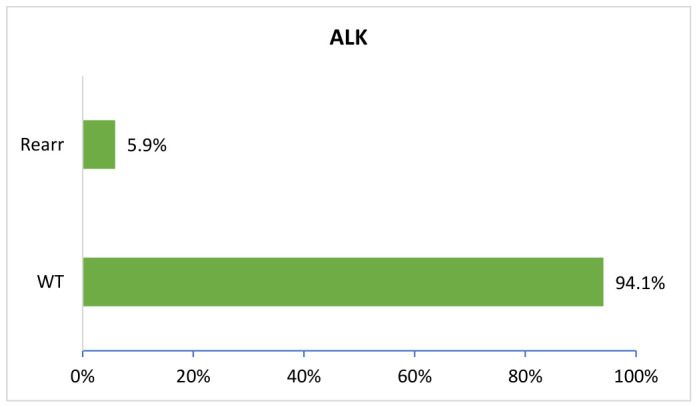
Distribution of *ALK* rearrangement status.

**Figure 12:**
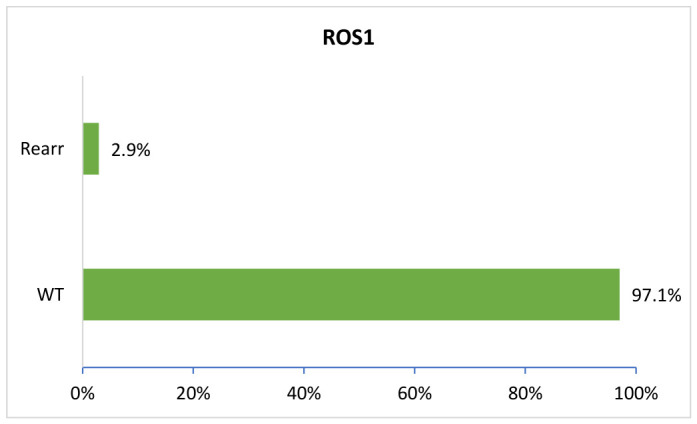
Distribution of *ROS1* rearrangement status.

**Figure 13:**
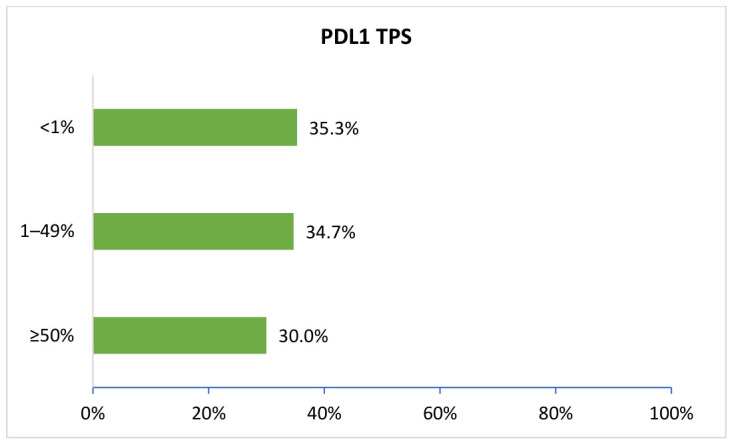
Distribution of PD-L1 tumor proportion score categories.

The molecular profile of the tumors of these patients was studied. *EGFR* mutations were detected in 32 patients (18.8%), while the remaining 138 (81.2%) were wild-type (Figure 8). *KRAS* mutations were detected in 33 patients (19.4%), while 137 patients (80.6%) were wild-type (Figure 9). *BRAF* mutations were found in seven patients (4.1%), while 163 patients (95.9%) were negative for this mutation (Figure 10). *ALK* rearrangements were detected in 10 patients (5.9%) (Figure 11), and *ROS1* rearrangements were detected in five patients (2.9%), while for both markers, most patients were wild-type (Figure 12). The three PD-L1 expression categories had a relatively uniform distribution. Sixty patients (35.3%) showed expression of <1%, and 59 patients (34.7%) showed expression of 1–49%, while 51 patients (30.0%) showed high PD-L1 expression of ≥50% (Figure 13).

**Figure 14:**
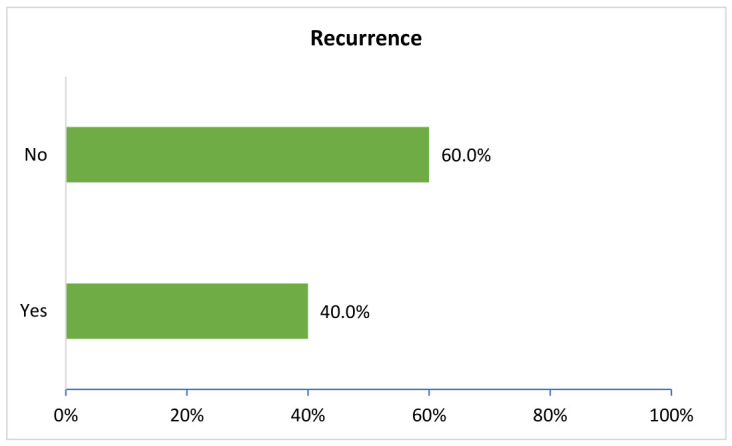
Distribution of disease relapse during follow-up.

**Figure 15:**
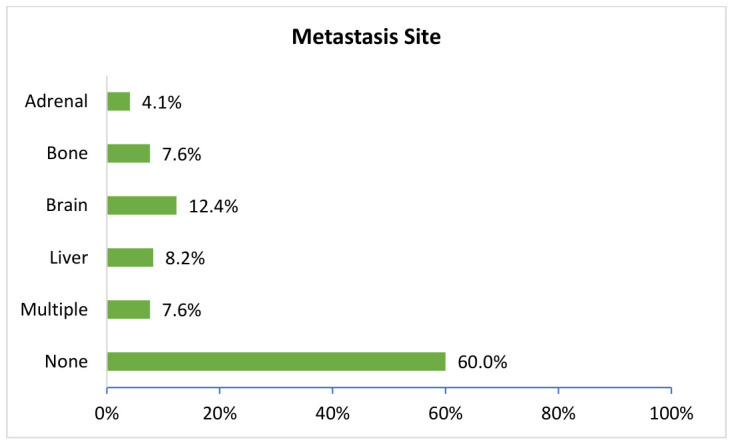
Distribution of metastatic sites among patients who developed disease relapse.

Disease relapse during follow-up was also recorded. Relapse occurred in 68 patients (40.0%), while 102 patients (60.0%) did not relapse (Figure 14). Among patients who developed metastases, the brain was the most common metastatic site in 21 patients (12.4%), followed by the liver in 14 patients (8.2%) and the bones in 13 patients (7.6%). Multiple metastatic locations were present in 13 patients (7.6%), and finally, the adrenal glands were involved in seven patients (4.1%) (Figure 15).

Finally, for the 68 patients who developed metastasis, time to metastasis was also analyzed. The mean time to metastasis was 16.97 months (SD: 11.34), with a median value of 15 months. The range of values was from 2 to 50 months (Table 1).

A comparison of pack years was performed between the groups that showed *EGFR*, *KRAS*, *BRAF* and *ALK* mutations, *ROS1* rearrangements and the corresponding wild-type groups. No statistically significant differences were observed (all *p* > 0.05). These findings were confirmed by the non-parametric Mann–Whitney U test. In the *ALK* rearrangement group, lower pack-years were recorded, suggesting a trend toward lower pack-year exposure compared with the wild-type group. However, statistical significance was not achieved (Table 2).

The possible association between smoking history and PD-L1 expression, as reflected by the Tumor Proportion Score (TPS), was investigated using correlation matrix analysis. It was found that there was no statistically significant association between PD-L1 expression (TPS) and smoking history. In the analysis with three categories of smoking status (current, former, and never), never smokers showed a descriptively higher percentage of PD-L1 TPS of ≥50% compared with the other groups; however, the difference was not statistically significant (χ^2^(4) = 2.806, *p* = 0.591, and Cramer’s V = 0.091) (Table 3). Similarly, in the dichotomous analysis (never vs. yes), never smokers more often showed high PD-L1 expression, but without a statistically significant difference (χ^2^(2) = 1.743, *p* = 0.418, and Cramer’s V = 0.101). Therefore, in this sample, smoking history did not appear to be significantly associated with the distribution of PD-L1 TPS (Table 4).

To investigate the factors independently associated with SUVmax, stepwise linear regression was applied with the variables gender, age, pack-years, corrected calcium, *EGFR*, *KRAS*, *BRAF*, *ALK*, *ROS1* and PDL1 TPS. The final model was statistically significant (F(2, 167) = 10.86 and *p* < 0.001, with R = 0.339, R^2^ = 0.115 and adjusted R^2^ = 0.104), indicating that it explains approximately 11.5% of the variance in SUVmax. Two variables as independent predictors remained in the final model. One of these was corrected calcium, which was positively and significantly associated with SUVmax (B = 2.765, β = 0.307, and *p* < 0.001), and it emerged as the strongest predictor in the model.

In addition, pack-years also showed a positive and statistically significant correlation with SUVmax (B = 0.062, β = 0.156, and *p* = 0.034).

In contrast, the variables gender, age, *EGFR*, *KRAS*, *BRAF*, *ALK*, *ROS1* and PDL1 TPS were not retained in the final model.

The regression equation was as follows:

SUVmax = −17.37 + 2.765 × Corrected Calcium + 0.062 × Pack Years

A Durbin–Watson value of 1.851 indicated the absence of significant autocorrelation of the residuals. However, normality tests of the residuals (Kolmogorov–Smirnov *p* = 0.015; Shapiro–Wilk *p* < 0.001) indicated a deviation from a normal distribution. Therefore, although the model was statistically significant, its explanatory power was relatively limited, and the results should be evaluated with caution (Table 5).

**Figure 16:**
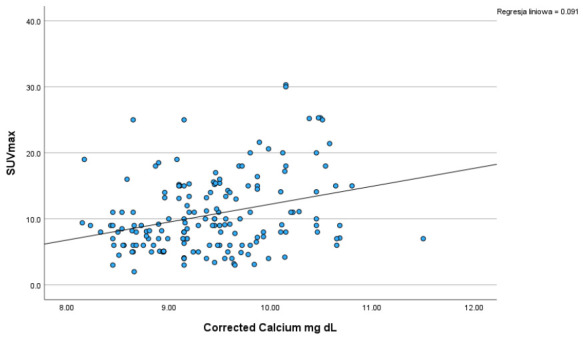
Correlation between corrected serum calcium levels and primary tumor SUVmax. Dots represent individual patients, and the line represents the fitted linear trend.

There was a statistically significant correlation between corrected calcium and SUVmax. The correlation coefficient was r = 0.301, with *p* = 0.0000647, in 170 patients (Figure 16) (Table 6). This indicates a positive association between the two variables; that is, as the values of corrected calcium increased, there was a tendency for SUVmax to increase. Although modest in magnitude, this correlation was statistically significant. In contrast, no statistically significant correlation of SUVmax with age, pack-years or time to metastasis was observed (Table 6).

The relationship of SUVmax with demographic, clinical and molecular factors was investigated using the independent-samples *t*-test. The results showed that no statistically significant differences were observed in SUVmax values with respect to gender (*p* = 0.957), *KRAS* (*p* = 0.206), *BRAF* (*p* = 0.858), *ALK* (*p* = 0.458), *ROS1* (*p* = 0.509), disease recurrence (*p* = 0.608) and smoking (*p* = 0.940) (Table 7).

**Figure 17:**
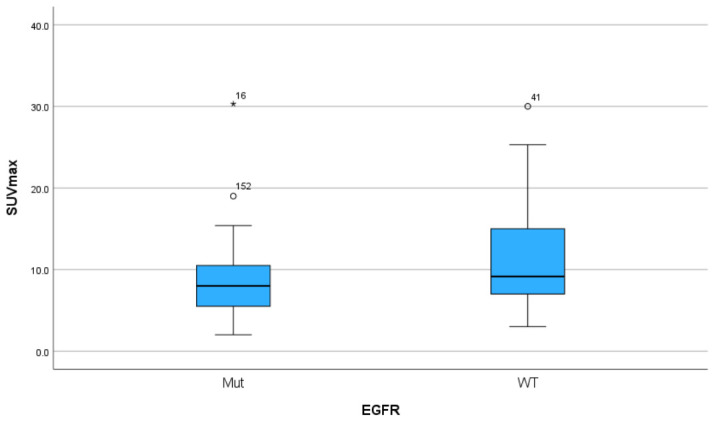
Comparison of SUVmax between *EGFR*-mutated and *EGFR* wild-type tumors. Boxes represent the interquartile range, horizontal lines represent medians, whiskers represent the data range, circles indicate outliers, the asterisk indicates an extreme outlier, and numbers indicate case identifiers.

However, a marginally non-statistically significant difference (*p* = 0.072) was recorded regarding *EGFR*; patients with wild-type *EGFR* seemed to have higher SUVmax values (mean = 11.00; SD = 5.74) compared with patients with mutated *EGFR* (mean = 8.97; SD = 5.57). The effect size for this comparison was small to moderate (Cohen’s d = −0.356). These findings indicate that for most of the factors examined, SUVmax did not appear to differ significantly, with the only notable finding being a trend toward an association with *EGFR* status (Figure 17).

**Figure 18:**
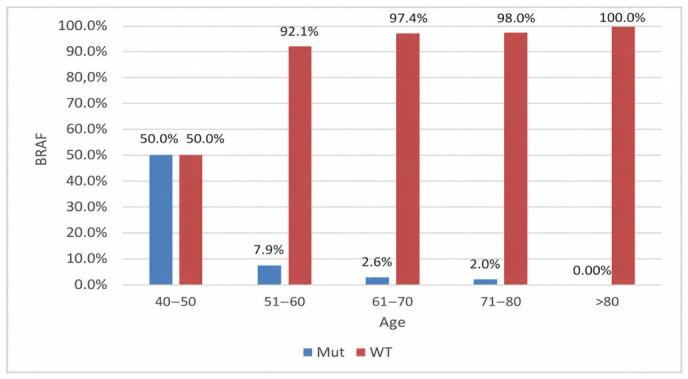
Distribution of *BRAF* mutation status across age groups.

Cross-tabulations were used to investigate the association between age and molecular/clinicopathological characteristics. The analysis showed a statistically significant correlation with age distribution, observed only for the *BRAF* variable (Pearson χ^2^, *p* = 0.010; exact test, *p* = 0.039; Cramer’s V = 0.278). This shows that the *BRAF* mutation was more common in younger patients. *BRAF* wild-type status was more common in older patients (Figure 18).

Age was not significantly associated with *EGFR* (*p* = 0.640), *KRAS* (*p* = 0.345), *ALK* (*p* = 0.706), *ROS1* (*p* = 0.903), PDL1 TPS (*p* = 0.419) and smoking status (*p* = 0.593) (Table 8, Table 9, Table 10, Table 11, Table 12, Table 13 and Table 14). Therefore, these findings in our specific sample show that age does not appear to significantly differ across most of the examined molecular and clinical characteristics, with the exception of *BRAF*. These results should be interpreted with caution because in the extreme age groups, we had a limited number of patients, and this may affect the statistical power of the analysis.

Therefore, these findings in our specific sample show that age does not appear to differ significantly across most of the examined molecular and clinical characteristics, with the exception of *BRAF*. These results should be interpreted with caution because in the extreme age groups, we had a limited number of patients, and this may affect the statistical power of the analysis.

## 4. Discussion

In the present study, the main findings were that corrected serum calcium and pack-years remained the only factors independently associated with SUVmax in the final linear regression model, whereas most of the molecular variables examined were not retained in the final model. In addition, corrected calcium showed a statistically significant positive correlation with SUVmax. These findings indicate that, in this particular sample, simple preoperative biochemical and clinical parameters were more strongly related to the metabolic activity of the tumor than the molecular markers included in the analysis.

Beyond the statistical associations observed in this cohort, these findings may have biological and translational relevance. SUVmax reflects, at least in part, tumor glucose utilization and metabolic activity, whereas corrected serum calcium and smoking exposure may represent broader systemic and tumor-related biological processes. The association between corrected calcium and SUVmax may point toward interactions between systemic metabolic status, calcium-dependent signaling, tumor aggressiveness, and intratumoral glucose metabolism. Similarly, the limited ability of smoking history alone to discriminate PD-L1 expression or molecular status suggests that clinical smoking variables may not fully capture the underlying genomic, inflammatory, and immune microenvironmental effects of tobacco exposure. Therefore, the revised Discussion aims to place the present findings in a broader biological and translational context, while avoiding causal interpretation beyond the retrospective observational data.

A particularly important finding of the present analysis was the relationship between corrected calcium and SUVmax. Corrected calcium showed a positive correlation with SUVmax and remained the strongest factor in the final regression model. This means that higher corrected calcium values were associated with higher metabolic activity of the primary tumor, as expressed by SUVmax. However, the strength of the correlation was moderate, suggesting that this specific parameter probably reflects only part of the biological behavior of the tumor and cannot be considered sufficient as a standalone marker [16,17]. Several biological mechanisms may potentially explain the observed association between corrected calcium levels and SUVmax. Calcium ions act as important intracellular second messengers and regulate multiple processes relevant to cancer biology, including mitochondrial function, ATP production, redox signaling, cellular proliferation, apoptosis resistance, angiogenesis, and adaptation to cellular stress [23,24]. Calcium-dependent signaling pathways are also closely linked to tumor metabolic reprogramming and may contribute to increased glycolytic activity and energetic demands in malignant cells [19]. Since FDG uptake reflects, at least in part, glucose utilization by metabolically active tumor cells, dysregulated calcium homeostasis could provide a plausible mechanistic link between higher corrected calcium levels and increased SUVmax. However, serum corrected calcium should not be interpreted as a direct measure of intracellular calcium signaling within the tumor. Higher systemic calcium levels may also reflect indirect tumor- or host-related processes, such as paraneoplastic activity, bone turnover, systemic inflammation, hypoxia-related metabolic adaptation, or more aggressive tumor behavior, all of which may themselves be associated with increased FDG uptake [17,25]. Therefore, the association observed in the present study should be regarded as hypothesis-generating rather than causal and may reflect a broader interaction between systemic metabolic status, tumor aggressiveness, and intratumoral metabolic activity. Future studies incorporating tissue-level calcium-signaling markers, inflammatory biomarkers, and broader molecular profiling are needed to further clarify this relationship.

Pack-years also remained in the final regression model and showed a positive relationship with SUVmax. In other words, greater cumulative smoking exposure was associated with higher SUVmax values in the context of the multivariable analysis. Nevertheless, in the simple correlation analysis, no statistically significant correlation between pack-years and SUVmax was documented. This finding suggests that the effect of pack-years probably becomes evident mainly within the multivariable model and not as a strong individual linear relationship. Therefore, the contribution of smoking burden to the variability of SUVmax appears to be present but limited [7,8,13,14].

Despite the statistical significance of the final model, its explanatory ability was limited. Specifically, the model explained approximately 11.5% of the variance in SUVmax, which means that the greater part of the variability in tumor metabolic activity was not explained by the variables included. The limited explanatory ability of the regression model suggests that most of the variability in SUVmax is likely determined by factors not captured in the present analysis. These may include tumor-related characteristics such as tumor size, cellularity, histological grade, necrosis, hypoxia, proliferative activity, and intratumoral heterogeneity, as well as technical PET/CT-related parameters including uptake time, blood glucose levels, reconstruction methodology, and partial-volume effects [19,20]. Moreover, additional molecular alterations not assessed in this study, including TP53, STK11, KEAP1, MET, or HER2 alterations, and features of the tumor microenvironment, may also contribute to differences in FDG uptake. Thus, corrected calcium and pack-years should be interpreted as limited complementary indicators of tumor metabolic activity rather than as strong predictors of SUVmax. In addition, although the Durbin–Watson index did not indicate significant autocorrelation, the residuals did not follow a normal distribution. Therefore, the findings of the model should be interpreted with caution, as they demonstrate statistically significant associations, but do not support a particularly strong predictive model for SUVmax.

The direct comparisons of SUVmax among the demographic, clinical, and molecular subgroups were, for the most part, negative. No statistically significant differences in SUVmax were observed with respect to sex, *KRAS*, *BRAF*, *ALK*, *ROS1*, recurrence, or smoking. This pattern indicates that, in this particular sample, SUVmax did not substantially differ among most of the groups examined. The only exception was *EGFR*, for which a borderline, non-statistically significant trend toward higher SUVmax values was observed in wild-type tumors compared with *EGFR*-mutated ones [11,12,13,14]. Since this finding did not reach the predefined level of statistical significance, it should be regarded as a trend rather than as a confirmed association. Overall, the data of the present study do not support that SUVmax alone can clearly discriminate most of the molecular subgroups examined [11,12,13,14].

Regarding the relationship between smoking burden and molecular alterations, the analysis of pack-years did not reveal statistically significant differences between tumors with *EGFR*, *KRAS*, *BRAF*, *ALK*, or *ROS1* alterations and the corresponding wild-type groups. The same result was confirmed using the Mann–Whitney U test. Although descriptively tumors with *ALK* rearrangement showed lower pack-years, this difference was not statistically significant. Therefore, in the study sample, smoking burden did not appear to differ substantially among the molecular categories examined [3,7,8,10].

Similarly, the relationship between smoking and PD-L1 expression was not statistically significant. Neither the analysis using three categories of smoking status nor the dichotomous analysis showed a significant association with the distribution of PD-L1 TPS. Although descriptively, never-smokers more frequently showed PD-L1 TPS of ≥50%, the differences were not statistically significant, and the effect sizes were small. Consequently, based on the present data, smoking history did not appear to constitute a useful discriminating factor for PD-L1 expression in the present cohort [26,27,28,29]. This finding should be interpreted in the context of previous studies reporting an association between smoking exposure and increased PD-L1 expression in NSCLC. Smoking may promote genomic instability, increase tumor mutational burden, and induce inflammatory signaling pathways, all of which can contribute to immune checkpoint activation and PD-L1 upregulation [30,31]. However, clinical smoking history may not fully capture the biological consequences of tobacco exposure at the tumor level, including mutational signatures, DNA damage, tumor mutational burden, or the composition of the immune microenvironment. In addition, differences in disease stage, histological composition, molecular background, PD-L1 assay methodology, and sample size may partly explain discrepancies between studies. The present cohort consisted of surgically resected NSCLC cases, and subgroup sizes were limited for some molecular categories, which may have reduced the ability to detect modest associations between smoking history and PD-L1 TPS. Therefore, our findings do not exclude a biological link between smoking-related genomic instability and PD-L1 expression but suggest that clinical smoking history alone may be insufficient to predict PD-L1 expression in this cohort.

Age was not statistically significantly associated with *EGFR*, *KRAS*, *ALK*, *ROS1*, PD-L1 TPS, or smoking status. The only statistically significant association noted was for *BRAF*, with the *BRAF* mutation being more common in the younger patient group and *BRAF* wild type being more common in the older age group. However, the text describes the low number of patients in the age extremes, which may have influenced the results. Thus, this association is interesting in this series but should be interpreted with caution. With the exception of *BRAF*, age did not seem to play a significant role in discriminating among other variables. Because the number of patients with *BRAF*-mutated tumors was small, the association between younger age and *BRAF* mutation should be considered exploratory. In the present cohort, *BRAF*-mutated cases did not show a clearly distinct clinical disease course compared with older *BRAF* wild-type patients. No statistically significant difference in SUVmax was observed according to *BRAF* status, and *BRAF* mutation was not retained in the final regression model for SUVmax. In addition, the available data did not demonstrate a consistent pattern, suggesting that younger *BRAF*-mutated patients had a distinct relapse behavior or a clearly different clinical course. Therefore, although the age-related association with *BRAF* status is noteworthy, it should not be interpreted as evidence of a separate clinicopathological phenotype in this cohort. Larger studies with detailed assessment of *BRAF* mutation subtype, histological pattern, co-mutations, treatment exposure, and longitudinal outcomes are needed to clarify whether younger patients with *BRAF*-mutated NSCLC represent a biologically distinct subgroup [32,33].

Overall, the results of the present study show that corrected calcium and pack-years were the only factors retained as independent indicators of SUVmax, whereas most molecular variables were associated neither with significant differences in SUVmax nor with retention in the final model. At the same time, no significant differences in pack-years were found among the molecular groups examined, nor was a significant association of smoking with PD-L1 TPS identified. In addition, age did not appear to differentiate most variables, with the exception of *BRAF*. These findings suggest that, in this particular cohort of patients with resected NSCLC, simple preoperative clinical and biochemical parameters did provide some information regarding the metabolic activity of the tumor, but their relationship with the individual molecular categories was generally limited and, in most analyses, not statistically significant.

From a clinical perspective, the present findings suggest that simple preoperative clinical and biochemical parameters may provide complementary information regarding tumor metabolic activity in surgically resectable NSCLC. Corrected serum calcium and cumulative smoking exposure are routinely available before surgery and may help contextualize SUVmax findings as part of a broader preoperative assessment. However, these parameters should not be used as standalone biomarkers for predicting molecular alterations, PD-L1 expression, or postoperative disease course. Instead, they may be considered supportive variables that reflect aspects of systemic metabolic status, smoking-related exposure, and tumor biology. Their potential clinical value lies mainly in complementing, rather than replacing, established imaging, histopathological, and molecular evaluation. At the same time, the clinical applicability of these findings remains limited by the retrospective single-center design and the modest explanatory power of the regression model. In this context, previous work has emphasized that NSCLC progression is clinically heterogeneous and may include different patterns, such as synchronous lymph node involvement, local or regional recurrence, and distant metastasis, with different prognostic implications [2]. This supports the need for integrative risk assessment models that combine routinely available clinical and biochemical variables with imaging-derived parameters and molecular data. Therefore, future prospective and multicenter studies are needed to validate whether the integration of corrected calcium, smoking exposure, PET/CT parameters, radiomic features, and broader multi-omics data, including genomic, transcriptomic, proteomic, and metabolomic profiles, can improve preoperative risk stratification, prediction of tumor biology, or individualized follow-up strategies in patients with resectable NSCLC [34,35].

**Figure 19:**
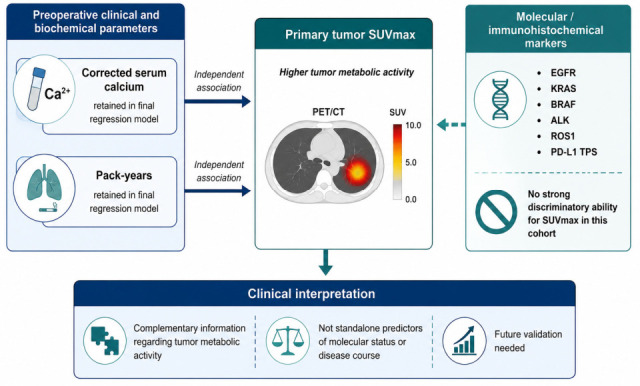
Graphical summary of the main findings. Corrected serum calcium and cumulative smoking exposure, expressed as pack-years, were retained in the final regression model as independent indicators of primary tumor SUVmax. In contrast, the examined molecular and immunohistochemical markers, including *EGFR*, *KRAS*, *BRAF*, *ALK*, *ROS1*, and PD-L1 TPS, did not show strong discriminatory ability for SUVmax in this cohort. Overall, the findings suggest that routinely available preoperative clinical and biochemical parameters may provide complementary information regarding tumor metabolic activity, but should not be interpreted as standalone predictors of molecular status or disease course. Future validation in larger cohorts is required. Solid arrows indicate observed associations retained in the regression model, whereas dashed arrows indicate limited or non-discriminatory relationships with molecular/immunohistochemical markers in this cohort.

The present study has certain limitations that arise directly from the analyses presented. First, the final regression model had limited explanatory ability. Second, the deviation of the residuals from normality suggests that the model must be interpreted with caution. Third, several subgroup analyses were negative, while for certain variables, the text itself points out a small number of patients in specific categories, especially at the extremes of age. In particular, some molecular subgroups, such as *BRAF*-mutated, *ALK*-rearranged, and *ROS1*-rearranged tumors, included a limited number of patients. This reduced the statistical power to detect modest differences between groups and increased the risk of unstable estimates in subgroup comparisons. In small datasets, regression-type models and subgroup analyses may be particularly vulnerable to overfitting and unreliable estimates when the number of predictors or comparisons is high relative to the number of observations [36]. Therefore, the negative findings observed in these subgroups should not be interpreted as definitive evidence of the absence of association. Rather, they should be considered exploratory and hypothesis-generating, requiring confirmation in larger, preferably multicenter cohorts with adequate representation of rare molecular alterations. In addition, although several clinicopathological variables such as histological subtype, pathological stage, tumor location, and relapse status were available, we did not further expand the multivariable regression model or perform extensive stratified analyses because of the limited sample size and the relatively small number of patients in several molecular subgroups. Including a larger number of covariates in this setting could have increased the risk of model overfitting and unstable estimates. Therefore, the present analysis should be interpreted as exploratory, and future larger multicenter studies should evaluate whether the association between corrected calcium, smoking exposure, and SUVmax remains consistent after stratification by histology, pathological stage, and other clinicopathological variables. These points mean that the absence of statistically significant differences in several comparisons should not be interpreted absolutely. Nevertheless, the analysis consistently identified corrected calcium and pack-years as the only factors retained in the final model for SUVmax. It should also be emphasized that the findings directly derived from the present cohort should be distinguished from literature-based biological interpretations. The direct results of the present study demonstrate associations between corrected calcium, pack-years, and SUVmax, as well as the absence of statistically significant associations between most molecular subgroups and SUVmax. In contrast, the proposed biological explanations regarding calcium signaling, tumor glucose metabolism, smoking-related genomic instability, PD-L1 expression, and *BRAF*-mutated NSCLC are interpretative and based on previously published literature. Therefore, these mechanistic considerations should be viewed as literature-supported interpretations that provide biological context for the observed associations and warrant further validation in future studies. A graphical summary of the main findings is presented in Figure 19.

## 5. Conclusions

The findings of the present study show that corrected serum calcium was positively associated with SUVmax and constituted the strongest factor in the final linear regression model, while pack-years also remained independently associated with SUVmax. In contrast, no significant associations were found between smoking history and PD-L1 TPS, no significant differences in pack-years were observed among the molecular groups examined, and SUVmax did not differ significantly across most molecular subgroups. The only individual observations were the non-statistically significant trend toward lower SUVmax in *EGFR*-mutated tumors [11,12,13,14] and the significant association of a younger age profile with *BRAF* mutation. Overall, in this particular sample, preoperative clinical and biochemical parameters appeared to be more closely related to the metabolic activity of the tumor than to a clear separation of the individual molecular categories.

## Figures and Tables

**Table 1:** Baseline demographic, clinical, metabolic, molecular, and follow-up characteristics of the study cohort.

Statistics						
		Age	Pack Years	SUVmax	Corrected Calcium mg dL	Time to Metastasis Months
N	Valid	170	170	170	170	68
	Missing	0	0	0	0	102
Mean		66.07	31.92	10.62	9.41	16.97
Median		66.00	36.00	9.00	9.39	15.00
Std. Deviation		7.52	14.44	5.75	0,64	11.34
Minimum		44.00	0.00	2.00	8.15	2.00
Maximum		85.00	62.00	30.30	11.50	50.00

**Table 2:** Comparison of smoking exposure, expressed as pack-years, according to molecular alteration status.

*t*-Test						Levene’s Test for Equality of Variances		*t*-Test for Equality of Means							Effect Sizes Cohen’s d	*p* (Mann-Whitney U)
						F	Sig.	*t*	df	*p*	Mean Difference	Std. Error Difference	95% Confidence Interval of the Difference			
		N	Mean	Std. Deviation	Std. Error Mean								Lower	Upper		
*EGFR*																
Pack Years	Mut	32	32.91	12.98	2.30	1.47	0.227	0.43	168.00	0.669	1.22	2.84	−4.39	6.82	0.084	0.697
	WT	138	31.69	14.79	1.26											
*KRAS*																
Pack Years	Mut	33	33.36	10.55	1.84	5.33	0.022	0.80	68.22	0.428	1.79	2.25	−2.70	6.29	0.124	0.744
	WT	137	31.57	15.24	1.30											
*BRAF*																
Pack Years	Mut	7	33.71	15.54	5.87	0.04	0.839	0.34	168.00	0.738	1.87	5.59	−9.16	12.90	0.129	0.441
	WT	163	31.84	14.44	1.13											
*ALK*																
Pack Years	Rearr	10	22.30	19.49	6.16	6.50	0.012	−1.63	9.58	0.135	−10.22	6.26	−24.25	3.82	−0.716	0.123
WT	160	32.52	13.92	1.10												
*ROS1*																
Pack Years	Rearr	5	30.00	17.49	7.82	0.13	0.720	−0.30	168.00	0.764	−1.98	6.57	−14.95	11.00	−0.136	0.806
WT	165	31.98	14.40	1.12												

**Table 3:** Association between smoking status and PD-L1 tumor proportion score categories.

Crosstab						
			Smoking Status			Total
			Current	Former	Never	
PDL1 TPS	<1%	N	17	30	13	60
		%	40.5%	34.9%	31.0%	35.3%
	1–49%	N	16	30	13	59
		%	38.1%	34.9%	31.0%	34.7%
	≥50%	N	9	26	16	51
		%	21.4%	30.2%	38.1%	30.0%
Total		N	42	86	42	170
		%	100.0%	100.0%	100.0%	100.0%

Cramer’s V = 0.091, Chi-Square Tests = 2.806, df = 4, *p* Asymptotic Significance = 0.591, *p* Exact Significance = 0.600.

**Table 4:** Association between never-/ever-smoking status and PD-L1 tumor proportion score categories.

Crosstab					
			Smoking Status		Total
			Never	Yes	
PDL1 TPS	<1%	N	13	47	60
		%	31.0%	36.7%	35.3%
	1–49%	N	13	46	59
		%	31.0%	35.9%	34.7%
	≥50%	N	16	35	51
		%	38.1%	27.3%	30.0%
Total		N	42	128	170
		%	100.0%	100.0%	100.0%

Cramer’s V = 0.101, Chi-Square Tests = 1.743, df = 2, *p* Asymptotic Significance = 0.418, *p* Exact Significance = 0.447.

**Table 5:** Stepwise linear regression model evaluating independent predictors of primary tumor SUVmax.

Dependent Variable: SUVmax						
Model Summary						
Model	R	R Square	Adjusted R Square	Durbin-Watson	F	Sig.
Regression	0.339	0.115	0.104	1.851	10.857	<0.001
Predictors: (Constant), Corrected Calcium mg dL, Pack Years						
Dependent Variable: SUVmax						
Coefficients						
Model		Unstandardized Coefficients		Standardized Coefficients	*t*	Sig.
		B	Std. Error	Beta		
Regression	(Constant)	−17.372	6.290		−2.762	0.006
	Corrected Calcium mg dL	2.765	0.656	0.307	4.212	0.000
	Pack Years	0.062	0.029	0.156	2.136	0.034
Dependent Variable: SUVmax						
Excluded Variables						
Model		Beta In	*t*	Sig.	Partial Correlation	Collinearity Statistics
						Tolerance
Regression	Sex	0.024	0.330	0.742	0.026	0.993
	Age	0.025	0.343	0.732	0.027	0.994
	*EGFR*	0.131	1.813	0.072	0.139	0.997
	*KRAS*	0.099	1.359	0.176	0.105	0.997
	*BRAF*	−0.016	−0.215	0.830	−0.017	0.999
	*ALK*	−0.075	−1.017	0.311	−0.079	0.971
	*ROS1*	0.023	0.318	0.751	0.025	0.993
	PDL1 TPS	0.001	0.012	0.991	0.001	0.981

Predictors in the Model: (Constant), Corrected Calcium mg dL, Pack Years.

**Table 6:** Correlation analysis between SUVmax and continuous clinical, metabolic, and follow-up variables.

		SUVmax
Age	Pearson Correlation	0.037
	Sig. (2-tailed)	0.636
	N	170
Pack Years	Pearson Correlation	0.145
	Sig. (2-tailed)	0.059
	N	170
Corrected Calcium mg dL	Pearson Correlation	0.301 **
	Sig. (2-tailed)	0.000
	N	170
Time To Metastasis months	Pearson Correlation	−0.145
	Sig. (2-tailed)	0.239
	N	68

** Correlation is significant at the 0.01 level (2-tailed).

**Table 7:** Comparison of SUVmax according to demographic, clinical, and molecular characteristics.

Independent Samples Test															
*t*-Test															
*t*-Test						Levene’s Test for Equality of Variances		*t*-Test for Equality of Means							Effect Sizes Cohen’s d
						F	Sig.	*t*	df	*p*	Mean Difference	Std. Error Difference	95% Confidence Interval of the Difference		
		N	Mean	Std. Deviation	Std. Error Mean								Lower	Upper	
Sex															
SUVmax	Female	68	10.65	5.03	0.61	1.56	0.214	0.05	168	0.957	0.05	0.90	−1.73	1.83	0.009
	Male	102	10.60	6.20	0.61										
*EGFR*															
SUVmax	Mut	32	8.97	5.57	0.99	1.55	0.215	−1.81	168	0.072	−2.03	1.12	−4.24	0.18	−0.356
	WT	138	11.00	5.74	0.49										
*KRAS*															
SUVmax	Mut	33	9.48	4.75	0.83	3.71	0.056	−1.27	168	0.206	−1.41	1.11	−3.61	0.78	−0.246
	WT	137	10.89	5.95	0.51										
*BRAF*															
SUVmax	Mut	7	11.00	4.22	1.59	0.45	0.503	0.18	168	0.858	0.40	2.23	−3.99	4.79	0.069
	WT	163	10.60	5.81	0.46										
*ALK*															
SUVmax	Rearr	10	11.93	5.85	1.85	0.11	0.746	0.74	168	0.458	1.39	1.88	−2.31	5.10	0.242
	WT	160	10.54	5.75	0.45										
*ROS1*															
SUVmax	Rearr	5	8.94	7.28	3.25	0.37	0.546	−0.66	168	0.509	−1.73	2.61	−6.89	3.43	−0.300
	WT	165	10.67	5.72	0.44										
Recurrence															
SUVmax	No	102	10.80	6.24	0.62	2.44	0.120	0.51	168	0.608	0.46	0.90	−1.32	2.24	0.080
	Yes	68	10.34	4.96	0.60										
Smoking Status															
SUVmax	Never	42	10.56	5.36	0.83	1.84	0.177	−0.08	168	0.940	−0.08	1.03	−2.10	1.95	−0.013
	Yes	128	10.64	5.89	0.52										

**Table 8:** Association between age group and *EGFR* mutation status.

Crosstab								
			Age					Total
			40–50	51–60	61–70	71–80	>80	
*EGFR*	Mut	N	0	5	16	11	0	32
		%	0.0%	13.2%	20.5%	22.4%	0.0%	18.8%
	WT	N	2	33	62	38	3	138
		%	100.0%	86.8%	79.5%	77.6%	100.0%	81.2%
Total		N	2	38	78	49	3	170
		%	100.0%	100.0%	100.0%	100.0%	100.0%	100.0%

Cramer’s V = 0.122, Chi-Square Tests = 2.525, df = 4, *p* Asymptotic Significance = 0.640, *p* Exact Significance = 0.622.

**Table 9:** Association between age group and *KRAS* mutation status.

Crosstab								
			Age					Total
			40–50	51–60	61–70	71–80	>80	
*KRAS*	Mut	N	1	5	13	13	1	33
		%	50.0%	13.2%	16.7%	26.5%	33.3%	19.4%
	WT	N	1	33	65	36	2	137
		%	50.0%	86.8%	83.3%	73.5%	66.7%	80.6%
Total		N	2	38	78	49	3	170
		%	100.0%	100.0%	100.0%	100.0%	100.0%	100.0%

Cramer’s V = 0.162, Chi-Square Tests = 4.481, df = 4, *p* Asymptotic Significance = 0.345, *p* Exact Significance = 0.319.

**Table 10:** Association between age group and *BRAF* mutation status.

Crosstab								
			Age					Total
			40–50	51–60	61–70	71–80	>80	
*BRAF*	Mut	N	1	3	2	1	0	7
		%	50.0%	7.9%	2.6%	2.0%	0.0%	4.1%
	WT	N	1	35	76	48	3	163
		%	50.0%	92.1%	97.4%	98.0%	100.0%	95.9%
Total		N	2	38	78	49	3	170
		%	100.0%	100.0%	100.0%	100.0%	100.0%	100.0%

Cramer’s V = 0.278, Chi-Square Tests =13.178, df = 4, *p* Asymptotic Significance = 0.010, *p* Exact Significance = 0.039.

**Table 11:** Association between age group and *ALK* rearrangement status.

Crosstab								
			Age					Total
			40–50	51–60	61–70	71–80	>80	
*ALK*	Rearr	N	0	4	4	2	0	10
		%	0.0%	10.5%	5.1%	4.1%	0.0%	5.9%
	WT	N	2	34	74	47	3	160
		%	100.0%	89.5%	94.9%	95.9%	100.0%	94.1%
Total		N	2	38	78	49	3	170
		%	100.0%	100.0%	100.0%	100.0%	100.0%	100.0%

Cramer’s V = 0.113, Chi-Square Tests = 2.160, df = 4, *p* Asymptotic Significance = 0.706, *p* Exact Significance = 0.619.

**Table 12:** Association between age group and *ROS1* rearrangement status.

Crosstab								
			Age					Total
			40–50	51–60	61–70	71–80	>80	
*ROS1*	Rearr	N	0	2	2	1	0	5
		%	0.0%	5.3%	2.6%	2.0%	0.0%	2.9%
	WT	N	2	36	76	48	3	165
		%	100.0%	94.7%	97.4%	98.0%	100.0%	97.1%
Total		N	2	38	78	49	3	170
		%	100.0%	100.0%	100.0%	100.0%	100.0%	100.0%

Cramer’s V = 0.078, Chi-Square Tests = 1.047, df = 4, *p* Asymptotic Significance = 0.903, *p* Exact Significance = 0.753.

**Table 13:** Association between age group and PD-L1 tumor proportion score categories.

Crosstab								
			Age					Total
			40–50	51–60	61–70	71–80	>80	
PDL1 TPS	<1%	N	1	14	29	15	1	60
		%	50.0%	36.8%	37.2%	30.6%	33.3%	35.3%
	1–49%	N	0	14	27	16	2	59
		%	0.0%	36.8%	34.6%	32.7%	66.7%	34.7%
	≥50%	N	1	10	22	18	0	51
		%	50.0%	26.3%	28.2%	36.7%	0.0%	30.0%
Total		N	2	38	78	49	3	170
		%	100.0%	100.0%	100.0%	100.0%	100.0%	100.0%

Kendall’s tau-c = 0.054, *p* Asymptotic Significance = 0.419, *p* Exact Significance = 0.424, Cramer’s V = 0.114, Chi-Square Tests = 4.384, df = 8, *p* Asymptotic Significance = 0.821, *p* Exact Significance = 0.860.

**Table 14:** Association between age group and smoking status.

Crosstab								
			Age					Total
			40–50	51–60	61–70	71–80	>80	
Smoking Status	Current	N	0	12	18	10	2	42
		%	0.0%	31.6%	23.1%	20.4%	66.7%	24.7%
	Former	N	1	18	38	28	1	86
		%	50.0%	47.4%	48.7%	57.1%	33.3%	50.6%
	Never	N	1	8	22	11	0	42
		%	50.0%	21.1%	28.2%	22.4%	0.0%	24.7%
Total		N	2	38	78	49	3	170
		%	100.0%	100.0%	100.0%	100.0%	100.0%	100.0%

Cramer’s V = 0.138, Chi-Square Tests = 6.485, df = 8, *p* Asymptotic Significance = 0.593, *p* Exact Significance = 0.611.

## Data Availability

The data supporting the findings of this study are available from the corresponding author upon reasonable request.

## Abbreviations

The following abbreviations are used in this manuscript:

**Abbreviation**	**Definition**
NSCLC	Non-small-cell lung cancer
SUVmax	Maximum standardized uptake value
PET/CT	Positron emission tomography/computed tomography
18F-FDG	18F-fluorodeoxyglucose
*EGFR*	Epidermal growth factor receptor
*KRAS*	Kirsten rat sarcoma viral oncogene homolog
*BRAF*	B-Raf proto-oncogene
*ALK*	Anaplastic lymphoma kinase
*ROS1*	ROS proto-oncogene 1
PD-L1	Programmed death-ligand 1
TPS	Tumor proportion score
FFPE	Formalin-fixed paraffin-embedded
NGS	Next-generation sequencing
FISH	Fluorescence in situ hybridization
PCR	Polymerase chain reaction
AJCC	American Joint Committee on Cancer
WHO	World Health Organization
SD	Standard deviation
